# Distribution of ACTN3 R577X, MTHFR C677T, MTHFR A1298C, and MTRR A66G Polymorphisms in 564 School-Aged Children in Beijing: A Cross-Sectional Study

**DOI:** 10.3390/genes17080886

**Published:** 2026-07-29

**Authors:** Xinrui Peng, Wangxin Ding, Xin Kang, Yuhao Yan, Yanjing Chen, Yan Zhang

**Affiliations:** 1 Precision Omics Laboratory for Sport Medicine & Engineering, Sports & Medicine Integrative Innovation Center, Capital University of Physical Education and Sports, Beijing 100191, China; 24008071009@cupes.edu.cn (X.P.); 24008071012@cupes.edu.cn (W.D.); 25008071001@cupes.edu.cn (X.K.); yanyh0909@163.com (Y.Y.); 2 Precision Omics Laboratory for Sport Medicine & Engineering, Laboratory and State Owned Assets Management Division, Capital University of Physical Education and Sports, Beijing 100191, China

**Keywords:** school-aged children, ACTN3, MTHFR, MTRR, genetic polymorphism, cross-sectional study, folate metabolism, Hardy-Weinberg equilibrium, population genetics, genetic baseline data

## Abstract

Background: Beijing’s 2024 school physical education reforms have substantially increased activity demands on children, yet population-level genotype data for Chinese schoolchildren remain scarce. Objectives: To characterize ACTN3 R577X, MTHFR C677T, MTHFR A1298C, and MTRR A66G polymorphisms in 564 Beijing schoolchildren; test Hardy-Weinberg equilibrium (HWE); compare allele frequencies with East Asian reference populations; and perform multi-locus cumulative allele analysis across all four loci. Methods: Cross-sectional study of 564 primary and secondary school students (265 males, 299 females) in Beijing. Genotyping was performed by PCR and Sanger sequencing using saliva-derived DNA; the overall call rate was 100% (564/564). HWE was assessed using the exact test of Wigginton et al. Allele frequencies were compared with 1000 Genomes CHB (n=103) and gnomAD EAS (n≈2604) using chi-squared or Fisher’s exact tests. Cumulative variant allele counts (0–8) were computed across all four loci. Linkage disequilibrium (LD) and haplotype frequencies were estimated using the EM algorithm. Results: All four loci were in HWE (all p>0.05). Genotype frequencies were: ACTN3 R577X—RR 31.56%, RX 49.11%, XX 19.33%; MTHFR C677T—CC 18.62%, CT 46.99%, TT 34.40%; MTHFR A1298C—AA 71.81%, AC 26.42%, CC 1.77%; MTRR A66G—AA 56.21%, AG 35.82%, GG 7.98%. No significant sex differences were observed at any locus (all p>0.05). Allele frequencies were consistent with East Asian reference populations. Across all four loci, 87.23% of students carried ≥2 variant alleles; 29.26% carried ≥4. LD between MTHFR C677T and A1298C was moderate to strong ($\left|D^{\prime }\right|=0.97$, $r^{2}=0.23$). The double-mutant T-C haplotype was rare (frequency =0.002). Conclusions: This study provides genotype frequency data for exercise- and folate metabolism-related polymorphisms in a sample of Beijing schoolchildren. These descriptive frequency data establish a baseline for future genotype–phenotype association studies; however, the sample is limited to two schools in two districts of Beijing and does not represent the broader Chinese pediatric population. Studies incorporating fitness, biochemical, and dietary data are required before such genetic profiles can inform educational or clinical recommendations. Given the purely descriptive, cross-sectional nature of the study and the absence of phenotypic data, these genotype frequency data do not, on their own, provide a basis for individualized physical education programming or nutritional recommendations.

## 1. Introduction

### 1.1. Policy Background and Practical Challenges

In 2024, the Beijing Municipal Education Commission launched the “Eight Physical Education Measures” (Tiyu Batiao) and the Student Physical Fitness Enhancement Plan, mandating daily physical education classes, at least one hour of moderate-to-vigorous physical activity (MVPA), and compulsory ball-sport instruction across all grade levels [1,2].

The increased demands raise questions about inter-individual variability in physiological adaptation. Children differ markedly in their capacity to tolerate and recover from physical exertion, and unaccounted differences may elevate the risk of overtraining, injury, or inadequate recovery [3,4].

### 1.2. Genetic Basis of Inter-Individual Variability

Inter-individual differences in athletic performance, exercise adaptation, and metabolic efficiency have a well-established genetic component. Heritability estimates for athletic status range from 30% to 80% depending on the specific phenotype, and over 200 genetic polymorphisms have been associated with physical performance traits [5,6,7,8].

The **ACTN3 R577X** variant (rs1815739) is one of the most studied polymorphisms in exercise genomics. ACTN3 encodes α-actinin-3, a Z-disc protein expressed exclusively in fast-twitch (type II) muscle fibres and essential for rapid, forceful contractions [9]. The R577X variant (C-to-T, exon 16) introduces a premature stop codon producing no α-actinin-3 in XX homozygotes. Approximately 18% of Europeans and 25% of East Asians are XX [10]. RR carriers typically show superior sprint and power performance. The role of the XX genotype in endurance performance is less consistent: while some studies suggest XX carriers may exhibit enhanced endurance capacity [11], other large-scale investigations have found no association between the XX genotype and endurance performance [12], and a recent meta-analysis reported significant heterogeneity across studies [13]. Meta-analyses confirm the R-allele association with elite power athlete status [14,15,16].

Beyond exercise performance, folate-mediated one-carbon metabolism plays a critical role in energy production, nucleotide synthesis, and methylation reactions, all heightened during periods of increased physical activity [17,18,19]. The **MTHFR C677T** (rs1801133) and **MTHFR A1298C** (rs1801131) polymorphisms reduce enzymatic activity by 30–70% depending on genotype combination, leading to decreased conversion of dietary folate to its bioactive form (5-methyltetrahydrofolate) and consequent elevation of plasma homocysteine [20,21]. The **MTRR A66G** (rs1801394) polymorphism further compounds this metabolic bottleneck by impairing the regeneration of methionine synthase [22]. These polymorphisms are highly prevalent in East Asian populations: the MTHFR 677T allele frequency ranges from 30% to 45% in Han Chinese, substantially higher than in European populations (25–35%) [23,24].

### 1.3. Rationale and Study Objectives

Despite the extensive literature on these polymorphisms in adult athletic and clinical populations, data on their distribution in Chinese school-aged children are remarkably limited. Establishing normative frequency data in this age group provides a genetic baseline for future genotype–phenotype association studies and may support longitudinal gene–environment interaction analyses in the context of school-based physical activity. It should be emphasized that the present study is purely descriptive in nature and does not include any phenotypic, biochemical, or dietary measurements.

The present study aimed to: (1) characterize the genotype and allele frequency distributions of ACTN3 R577X, MTHFR C677T, MTHFR A1298C, and MTRR A66G in 564 school-aged children in Beijing; (2) test for deviations from Hardy-Weinberg equilibrium (HWE); (3) compare allele frequencies with East Asian reference populations (1000 Genomes CHB and gnomAD EAS); (4) evaluate sex-stratified genotype distributions; and (5) perform multi-locus cumulative allele analysis and linkage disequilibrium/haplotype analysis.

## 2. Methods

### 2.1. Study Design and Participants

This was a cross-sectional observational study conducted in Beijing, China. A total of 564 primary and secondary school students were recruited from two public schools in Beijing, one in Daxing District and one in Chaoyang District, spanning grades 4 through 9 (age range: 9–15 years). All participants were healthy volunteers with no known underlying diseases. All participants were Han Chinese. Inclusion criteria were: (1) currently enrolled as a student in grades 4–9 at one of the participating schools; (2) able to provide a saliva sample; and (3) written informed consent provided by a parent or legal guardian. Exclusion criteria were: (1) diagnosed genetic or metabolic disorders; (2) acute illness at the time of sample collection; and (3) inability to provide an adequate saliva sample.

### 2.2. Ethical Considerations

This study was conducted in accordance with the Declaration of Helsinki and was approved by the Institutional Review Board of Capital University of Physical Education and Sports (Approval No. 2023A078 and 2023A079). Written informed consent was obtained from the parents or legal guardians of all participating students, and participation was entirely voluntary. Additional assent was obtained from students aged 8 years and above.

### 2.3. Sample Collection and DNA Extraction

Non-invasive saliva samples (1–3 mL) were collected using a saliva collection kit (10070812222384, JD.com, Inc., Beijing, China). Participants refrained from eating, drinking, or brushing their teeth for at least 30 min prior to collection. Samples were collected between 8:00 AM and 11:00 AM to minimize diurnal variation. Genomic DNA was extracted using the Universal Genomic DNA Extraction Kit (MD0201C09, Weidu Biotechnology, Suzhou, China) on an automated liquid handling workstation (EosBot NUSampro 3200, Qingyuankaiwu, Beijing, China). DNA concentration and purity were assessed using a NanoDrop One spectrophotometer (Thermo Fisher Scientific, Waltham, MA, USA) and a Qubit 4.0 fluorometer (Thermo Fisher Scientific, Waltham, MA, USA), with an OD_260_/OD_280_ ratio of 1.7–2.0 considered acceptable. Extracted DNA was stored at −20 °C until genotyping.

### 2.4. Genotyping

Genotyping of all four polymorphisms was performed by real-time fluorescence quantitative PCR (qPCR) amplification followed by Sanger sequencing. Real-time qPCR was employed to screen for successful amplification based on normal amplification curves, thereby eliminating the need for gel electrophoresis; only samples with satisfactory amplification curves were submitted for Sanger sequencing.

Primer sequences are listed in Table 1.

PCR reactions were carried out in a total volume of 20 μL containing 2 μL of genomic DNA template, 2× Taq Pro Universal SYBR qPCR Master Mix (Q712, Vazyme, Nanjing, China), 0.2 μM of each primer, 0.2 μM dUTP, and 0.2 μM UDG enzyme. Reaction setup and dispensing were performed on an automated liquid handling workstation (EosBot NUSampro 3200, Qingyuankaiwu, Beijing, China). Real-time qPCR was conducted on a 96E Real-Time PCR System (Tianlong Technology, Xi’an, China). Thermal cycling conditions were: 25 °C for 10 min (UDG treatment); initial denaturation at 95 °C for 2 min; 40 cycles of 95 °C for 15 s, 60 °C for 15 s, and 72 °C for 30 s; final extension at 72 °C for 2 min; and hold at 12 °C for 10 min.

Amplification products were submitted directly for bidirectional Sanger sequencing (Sangon Biotech, Shanghai, China). Chromatograms were visually inspected by two independent investigators using Sequencher v5.4.6 (Gene Codes Corporation, Ann Arbor, MI, USA).

Genotypes were designated as follows: ACTN3 R577X—RR (CC), RX (CT), XX (TT) using amino acid nomenclature; MTHFR C677T—CC, CT, TT; MTHFR A1298C—AA, AC, CC; MTRR A66G—AA, AG, GG. For MTHFR A1298C, complementary-strand assay results (T/G) were converted to standard A/C nomenclature (TT→AA, TG→AC, GG→CC).

### 2.5. Genotyping Success Rate and Quality Control

The overall genotype call rate was 100% (all 564 included samples had complete data for all four loci). Of 635 saliva samples initially collected, 573 (90.2%) yielded at least one genotype call and 564 (88.8%) yielded complete genotype data for all four loci. The remaining 62 samples (9.8%) failed to produce any genotype call, primarily due to insufficient DNA quantity or quality, and nine additional samples yielded partial results (three of four loci) and were excluded from the final analysis. A random 10% subset of the 564 included samples was re-genotyped in duplicate, achieving 100% concordance. For Sanger sequencing, chromatograms were visually inspected by two independent investigators using Sequencher v5.4.6 (Gene Codes Corporation, Ann Arbor, MI, USA), and only unambiguous, clearly interpretable reads were accepted. Negative controls (no-template controls) were included in each PCR batch. All genotyping was performed in a professionally managed molecular biology laboratory with stringent quality control and biosafety standards.

### 2.6. Statistical Analysis

All statistical analyses were performed using R version 4.5.2 and SPSS version 27.0. Statistical significance was set at a two-sided α=0.05, with Bonferroni correction applied for multiple comparisons.

**Descriptive Statistics.** Genotype and allele frequencies were calculated as counts and percentages with 95% confidence intervals (CIs) using the Clopper-Pearson exact method.

**Hardy-Weinberg Equilibrium.** HWE was assessed for each locus using the exact test based on the method of Wigginton et al. [25]. p>0.05 was considered consistent with HWE.

**Comparison with Reference Populations.** Allele frequencies were compared with the 1000 Genomes Project Phase 3 Han Chinese in Beijing (CHB) population (n=103) and the Genome Aggregation Database (gnomAD) v3.1.2 East Asian (EAS) population (n≈2604), both accessed March 2026, using the $\chi^{2}$ test or Fisher’s exact test.

**Sex-Stratified Analysis.** Genotype and allele frequency distributions were compared between male and female participants using $\chi^{2}$ tests.

**Multi-Locus Cumulative Allele Analysis.** For all four loci (ACTN3 R577X, MTHFR C677T, MTHFR A1298C, and MTRR A66G), a cumulative variant allele count (0–8) was calculated. It should be noted that this multi-locus analysis combines loci from distinct biological domains—ACTN3, which influences muscle fiber composition, and MTHFR/MTRR, which are involved in folate-mediated one-carbon metabolism—and therefore currently lacks a strong and specific biological rationale. This analysis is purely descriptive and exploratory; to our knowledge, no validated classification scheme exists for multi-locus genotype combinations in pediatric populations, and these groupings should be interpreted solely as descriptive summaries of multi-locus genetic variation rather than as clinically or biologically meaningful composite scores. The variant alleles were: ACTN3 X, MTHFR 677T, MTHFR 1298C, and MTRR 66G. Participants were grouped into three strata based on allele count: 0–2 alleles, 3–5 alleles, and 6–8 alleles. These thresholds were selected as an exploratory descriptive framework.

**Linkage Disequilibrium and Haplotype Analysis.** Pairwise LD between MTHFR C677T and A1298C was assessed using Lewontin’s $D^{\prime }$ and $r^{2}$ (genetics package, R 4.5.2). Haplotype frequencies were estimated using the expectation-maximization (EM) algorithm (haplo.stats package, R 4.5.2).

### 2.7. Sample Size Considerations

With a sample size of 564, the study had >99% power to detect deviations from HWE for variants with minor allele frequencies (MAF) ≥ 0.10, and >80% power for MAF ≥ 0.02. For comparisons with the 1000 Genomes CHB population (n=103) and gnomAD EAS population (n≈2604), the study had >80% power to detect allele frequency differences of ≥6% and ≥4%, respectively, for variants with MAF ≥ 0.15.

### 2.8. Use of Generative Artificial Intelligence Tools

**Figure 1:**
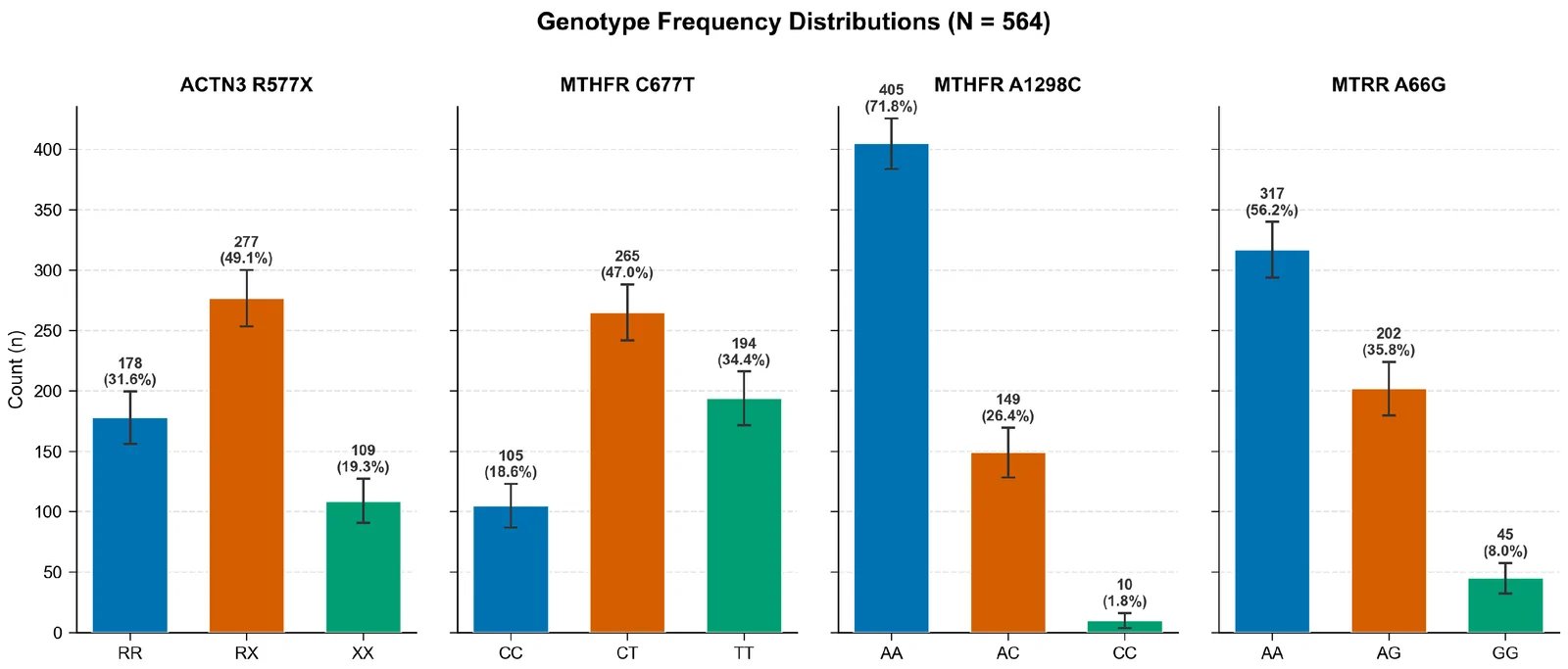
Genotype frequency distributions for the four polymorphisms in 564 Beijing schoolchildren. Bar charts show absolute counts and percentages for each genotype. Error bars represent 95% confidence intervals.

**Figure 2:**
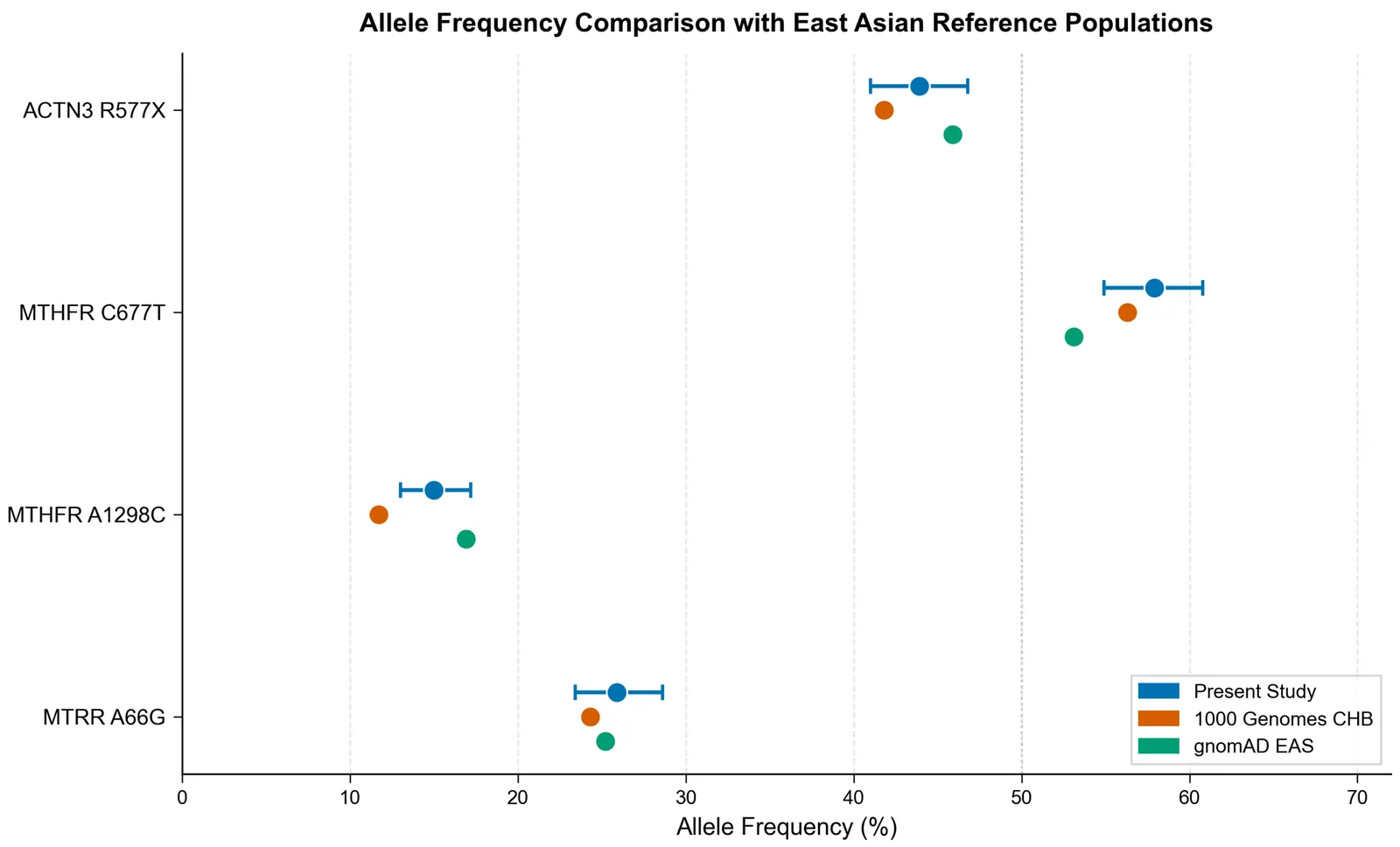
Allele frequency comparison between the present study and East Asian reference populations. Forest plot showing variant allele frequencies (with 95% CIs) for the present study, 1000 Genomes CHB (n=103), and gnomAD EAS (n≈2604).

**Figure 3:**
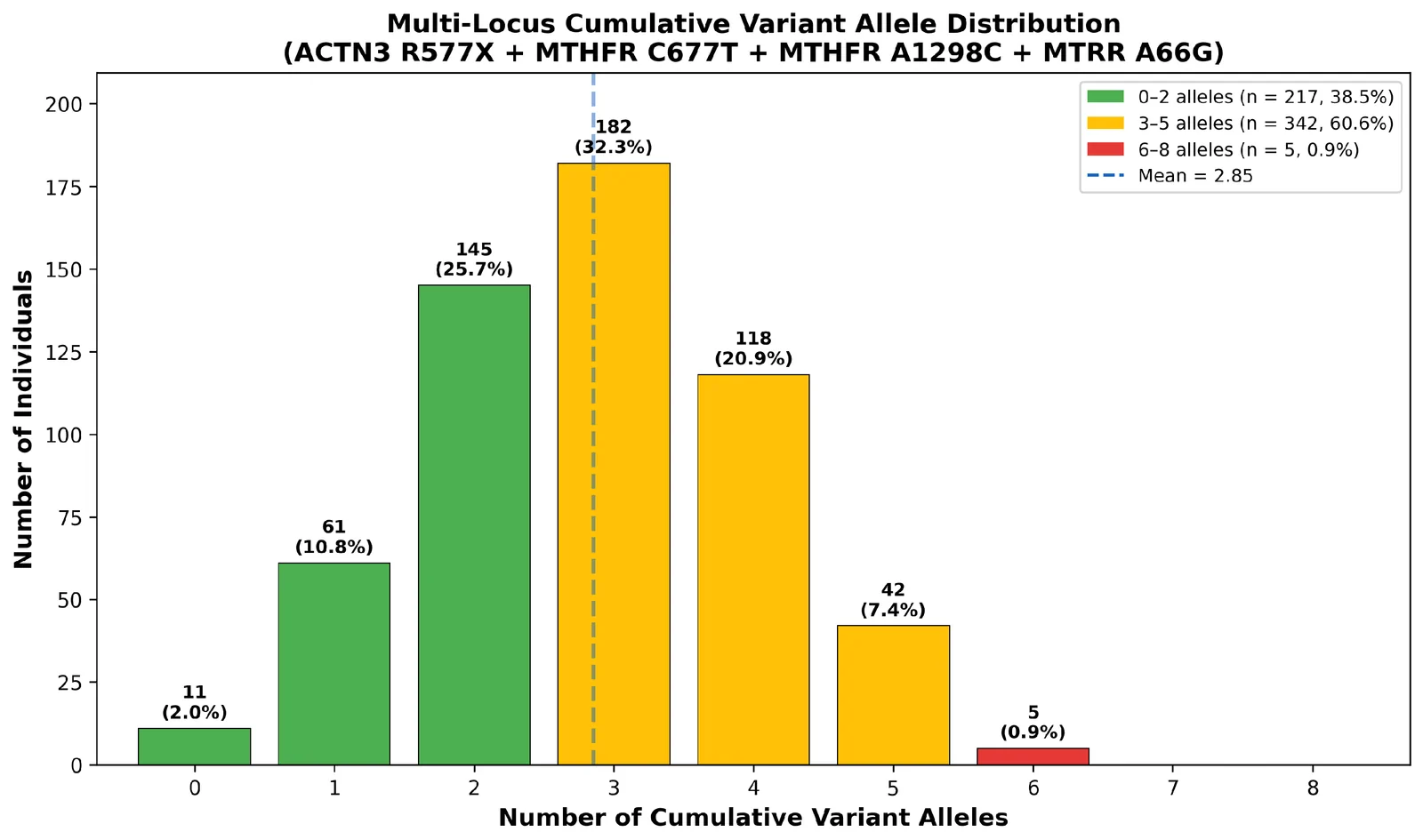
Multi-locus cumulative variant allele distribution across all four loci. Histogram showing the number of individuals carrying 0 through 8 cumulative variant alleles across ACTN3 R577X, MTHFR C677T, MTHFR A1298C, and MTRR A66G. Allele count groups are indicated by color: green (0–2 alleles), yellow (3–5 alleles), and red (6–8 alleles).

**Figure 4:**
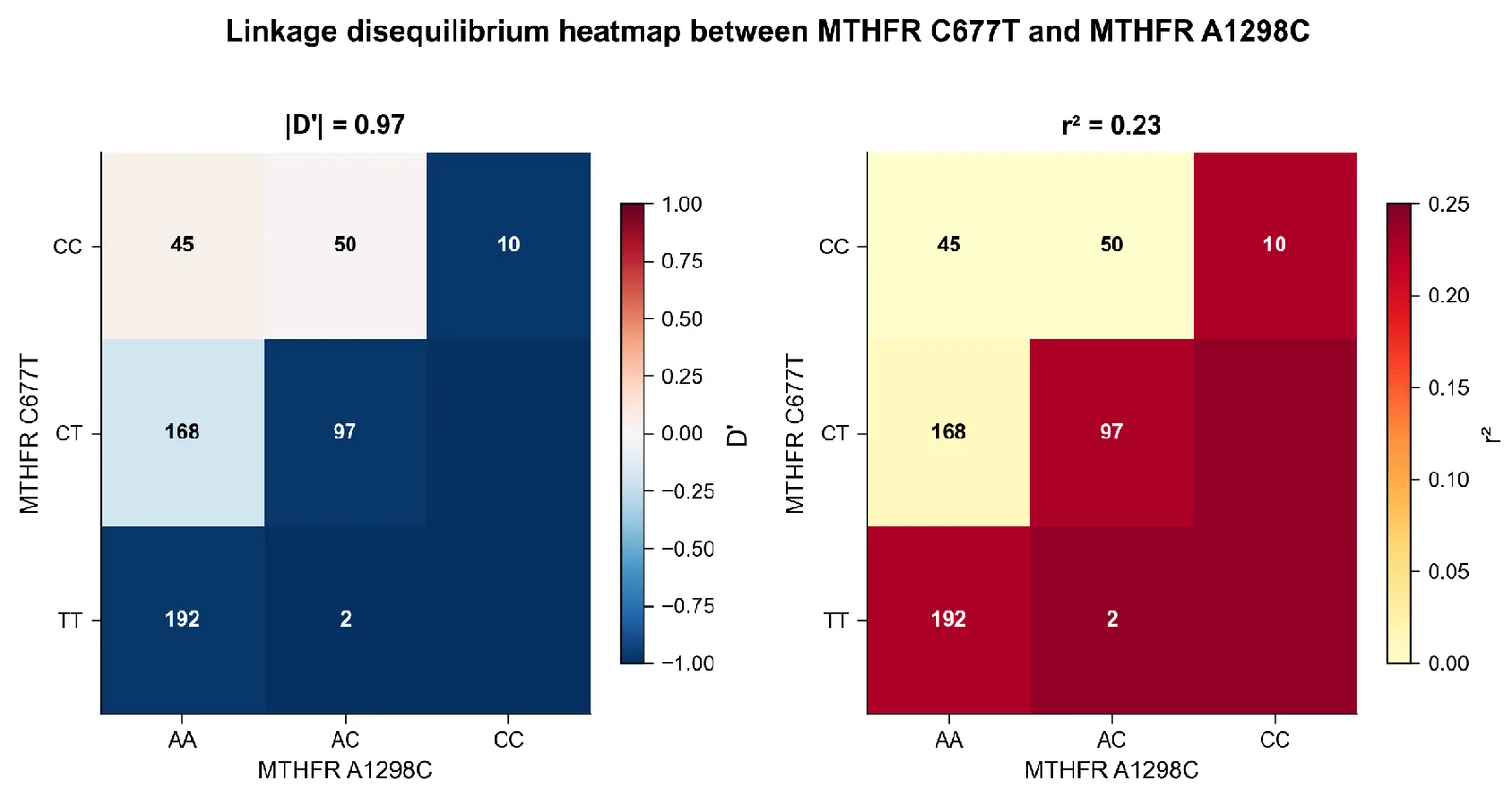
Linkage disequilibrium heatmap between MTHFR C677T and MTHFR A1298C. Color intensity represents the strength of LD as measured by $D^{\prime }$ and $r^{2}$.

During the preparation of this manuscript, generative artificial intelligence (GenAI) tools were used to assist in the creation of the graphical abstract. ChatGPT-4.0 (OpenAI, San Francisco, CA, USA) was used for text refinement of the graphical abstract, and Nanobanana was used for image generation. All other figures (Figure 1, Figure 2, Figure 3 and Figure 4) were generated using R 4.5.2.

## 3. Results

### 3.1. Participant Characteristics

A total of 564 school-aged children with complete genotype data for all four loci were included in the final analysis. The sample comprised 265 (46.99%) males and 299 (53.01%) females.

### 3.2. Genotype and Allele Frequency Distributions

Genotype and allele frequencies for all four loci are presented in Table 2 and Figure 1. All four loci were consistent with HWE (all p>0.05). The MTHFR 677T allele frequency was 57.89% (95% CI: 54.94–60.81%), with 34.40% of participants homozygous for the TT genotype. The ACTN3 X allele frequency was 43.88% (95% CI: 40.99–46.82%), with 19.33% XX homozygotes.

### 3.3. Sex-Stratified Genotype Distributions

Sex-stratified analyses revealed no statistically significant differences between males and females for any of the four loci (all p>0.05; Table 3). The largest absolute difference in allele frequency was observed for MTRR A66G (G allele: 24.72% vs. 26.92%, p=0.422).

### 3.4. Comparison with East Asian Reference Populations

Allele frequencies were compared with East Asian reference populations from the 1000 Genomes Project (CHB) and gnomAD (EAS) (Table 4, Figure 2). None of the four loci differed significantly from the CHB reference (all p>0.05). The MTHFR 677T allele showed a borderline difference against gnomAD EAS (57.89% vs. 53.10%, p=0.013; based on published frequencies), which did not survive Bonferroni correction (adjusted α=0.0125).

### 3.5. Multi-Locus Cumulative Variant Allele Distribution

The distribution of cumulative variant allele counts across all four loci is presented in Table 5 and Figure 3. The mean variant allele count was 2.85 ± 1.20 (range: 0–6). A total of 492 individuals (87.23%) carried two or more variant alleles, and 165 individuals (29.26%) carried four or more. A total of 217 individuals (38.48%) carried 0–2 variant alleles.

### 3.6. MTHFR Compound Genotypes, Linkage Disequilibrium, and Haplotypes

The compound genotype distribution for the two MTHFR loci is shown in Table 6. Compound heterozygosity at both loci (677CT + 1298AC) was observed in 97 individuals (17.20%). Homozygosity for both variant alleles (677TT + 1298CC) was not observed in any participant.

Linkage disequilibrium analysis between MTHFR C677T and MTHFR A1298C revealed $\left|D^{\prime }\right|=0.97$ (95% CI: 0.93–1.00) and $r^{2}=0.23$ (Table 7, Figure 4). Haplotype frequency estimation identified three major haplotypes: C-A (677C-1298A) at 27.4%, T-A (677T-1298A) at 57.6%, and C-C (677C-1298C) at 14.7%. The T-C double-mutant haplotype was rare (frequency =0.002), consistent with negative linkage disequilibrium (Table 8).

### 3.7. Multi-Locus Cumulative Variant Allele Distribution by Sex

The multi-locus cumulative variant allele group distribution was similar between males and females (Table 9, $\chi^{2}$ test, p=0.637).

Extended data, including multi-locus LD matrices, geographically referenced MTHFR C677T allele frequencies, and detailed sex-stratified analyses, are available in the Supplementary Materials (Tables S1–S5, Figure S1).

## 4. Discussion

### 4.1. Principal Findings

This cross-sectional study provides baseline genotype frequency data for four exercise- and folate-metabolism-related genetic polymorphisms in 564 school-aged children from two schools in two districts of Beijing. The principal findings are: (1) all four loci were in HWE, consistent with a randomly mating population free from significant selection or genotyping error; (2) the MTHFR 677T allele frequency (57.89%) is among the highest reported for any population; (3) the ACTN3 R577X genotype distribution reveals a diverse genotype distribution (RR 31.56%, RX 49.11%, XX 19.33%); (4) allele frequencies were broadly consistent with East Asian reference populations; and (5) 87.23% of participants carry at least two variant alleles across all four loci, while 29.26% carry four or more. It should be noted that the present study is limited to reporting genotype and allele frequency distributions; no phenotypic, biochemical, or dietary variables were measured, and the data therefore cannot support inferences about genotype–phenotype relationships or personalized exercise recommendations.

### 4.2. MTHFR C677T: High Prevalence in the Study Population

The MTHFR 677T allele frequency of 57.89% is markedly elevated compared with global averages and is consistent with previous reports in Northern Chinese populations [23,24]. With 34.40% of participants homozygous for the TT genotype—previously associated with approximately 30% residual MTHFR enzyme activity and reduced conversion of dietary folic acid to bioactive 5-methyltetrahydrofolate [20]—over one-third of the study population carries a genotype that may predispose to impaired folate metabolism. In adult populations, the TT genotype is associated with elevated plasma homocysteine and modestly increased cardiovascular risk [26].

The borderline significant difference in T allele frequency compared with gnomAD EAS (57.89% vs. 53.10%, p=0.013; based on published allele frequencies and may differ from exact database counts) may reflect genuine regional variation within East Asia, as the present sample is drawn from Northern China (Beijing), where MTHFR 677T allele frequencies have been reported to be higher than in Southern China and other East Asian regions [27]. Replication in larger, geographically diverse samples is warranted.

It is also noteworthy that allele frequencies for the other three loci (ACTN3 R577X, MTHFR A1298C, and MTRR A66G) showed no significant differences from East Asian reference populations, and no sex-specific differences were detected at any of the four loci examined. The absence of sex differences contrasts with some reports of sex-specific effects for MTHFR C677T in adult populations, which may reflect the prepubertal status of the present pediatric sample or insufficient statistical power to detect small effect sizes. These null findings are consistent with the expectation that autosomal genotype distributions are determined primarily by population-level genetic drift and migration patterns rather than by sex-specific selection, and they provide reassurance that sex-stratified analyses are not required for descriptive reporting of these loci in similar populations.

### 4.3. MTHFR A1298C and MTRR A66G: Compounding Folate Pathway Deficiencies

While MTHFR C677T has received the most attention in the literature, the contributions of MTHFR A1298C and MTRR A66G to the cumulative folate pathway genotype profile are noteworthy. The MTHFR 1298C allele frequency of 14.98% is consistent with previous reports in East Asian populations [24]. Although the homozygous CC genotype was rare (1.77%), the compound heterozygous state (677CT + 1298AC), observed in 17.20% of participants, is associated with enzyme activity reductions comparable to 677TT homozygosity (approximately 50–60% of normal) [28].

The MTRR 66G allele frequency of 25.89% adds a further dimension to the cumulative folate pathway genotype profile. The A66G variant (I22M) reduces enzyme activity by approximately 30–40%, impairing the regeneration of methionine synthase and consequently the remethylation of homocysteine to methionine [29]. In combination with MTHFR variants, MTRR A66G may further affect homocysteine metabolism when co-occurring with MTHFR variants. The multi-locus cumulative allele analysis across the three folate-pathway loci illustrates this point: while 70.21% of students carry at least two variant alleles across these three loci, the fact that only 4.79% carry four or more suggests that mildly to moderately reduced folate metabolism enzyme activity is the population norm, whereas the category with 4+ variant alleles remains relatively uncommon.

### 4.4. ACTN3 R577X: Population Distribution and Implications for Future Research

The ACTN3 R577X genotype distribution—RR 31.56%, RX 49.11%, XX 19.33%—reveals a genetically diverse genotype distribution. The X allele frequency of 43.88% is consistent with previous reports in Chinese populations and is higher than typically observed in West African populations but lower than in some European populations [10,30].

The functional significance of the R577X polymorphism is well-established. α-Actinin-3, encoded by the intact R allele, is a structural component of the Z-disc in fast-twitch (type II) muscle fibers and plays a critical role in anchoring actin filaments during rapid, forceful movements [9,31]. XX homozygotes exhibit a shift toward a more oxidative phenotype, with increased activity of enzymes associated with aerobic metabolism [11], which may underlie enhanced endurance performance and reduced susceptibility to exercise-induced muscle damage following eccentric contractions [32,33].

It must be emphasized that the present study is purely descriptive and cross-sectional in nature; no phenotypic data on physical fitness, exercise performance, muscle strength, or injury susceptibility were collected. The genotype frequency data reported here therefore cannot be used to infer how individual children may respond to physical activity or to guide physical education programming. Although the ACTN3 R577X polymorphism is one of the most extensively studied genetic variants in exercise genomics, the evidence linking genotype to real-world athletic performance—particularly endurance performance—remains inconsistent. For instance, while some studies have reported associations between the XX genotype and superior endurance capacity [11], a large-scale study of 698 Caucasian athletes found no association between ACTN3 R577X and endurance running times [12], and a recent meta-analysis documented significant heterogeneity across published studies [13]. Furthermore, athletic performance is a complex, polygenic trait influenced by numerous genetic variants, each with small effect sizes, as well as environmental, nutritional, and training factors [5,7,34]. The currently known genetic variants explain only a small fraction of the heritable component of athletic performance, and the use of DNA testing for talent identification or exercise prescription is not supported by current evidence [35,36,37]. These considerations underscore that the genotype frequency data reported here are purely descriptive and should be regarded solely as a baseline for future genotype–phenotype studies; they do not provide a basis for physical education programming or individual activity recommendations, which would require studies combining genetic data with actual physical fitness and performance phenotypes.

### 4.5. Integrating Multi-Locus Genotype Data

This study demonstrates the technical feasibility of simultaneously profiling multiple polymorphisms across distinct biological domains in a pediatric population, although the biological interpretation of combined genotypes from functionally unrelated loci requires considerable caution. The cumulative allele count, integrating information from four loci, reveals the distribution of variant alleles that would not be apparent from single-locus analysis alone. This descriptive multi-locus approach is consistent with population-based genetic profiling strategies [38,39], in which population-level genetic data may help frame hypotheses for future targeted research that includes phenotypic characterization. It must be emphasized, however, that the combination of the ACTN3 X allele with MTHFR and MTRR variants—given the different biological functions of these loci—is exploratory and currently lacks a specific biological rationale; the cumulative allele counts should therefore be interpreted solely as a descriptive summary of multi-locus genotype variation, not as a composite risk or trait score. Any practical implications of these genotype distributions can only be evaluated in studies that combine genetic data with actual fitness, biochemical, and dietary phenotypes.

### 4.6. Linkage Disequilibrium and Haplotype Analysis

The observed LD pattern between MTHFR C677T and A1298C ($\left|D^{\prime }\right|=0.97$, $r^{2}=0.23$) is consistent with published reports and reflects the well-documented negative association between the two variant alleles: the 677T allele almost never co-occurs *in cis* with the 1298C allele [40,41]. This was confirmed by the rare frequency (0.002) of the T-C haplotype and the absence of the 677TT + 1298CC genotype. The biological explanation likely involves negative selection against gametes carrying both mutations [42]. The $r^{2}$ value indicates that significant residual variation at each locus remains unexplained by the other, supporting genotyping of both loci for comprehensive MTHFR assessment.

### 4.7. Limitations

Several limitations should be considered when interpreting these results. First, this is a cross-sectional descriptive study; no phenotypic data (physical fitness, sports performance, body composition), biochemical parameters (homocysteine, folate, vitamin B12, hematological data), or dietary intake data were collected, precluding genotype–phenotype association analyses. Second, and critically, the sample was drawn from only two schools in two districts of a single city (Beijing) and exclusively comprised Han Chinese participants; the findings should therefore not be interpreted as representative of the broader Chinese pediatric population, which encompasses considerable geographic, ethnic, and socioeconomic diversity. The allele frequencies reported here may not generalize to other regions of China or to non-Han ethnic groups. In particular, MTHFR 677T allele frequencies are known to be substantially lower in Southern China [27], and the genetic profiles documented here reflect a specific Northern Chinese urban pediatric sample rather than the Chinese school-aged population as a whole. Third, the study was limited to four polymorphisms; athletic performance and metabolic capacity are polygenic traits influenced by numerous genetic variants, environmental factors, and gene–environment interactions, and the explanatory power of these four loci alone is modest. Fourth, the cross-sectional design precludes causal inference or longitudinal assessment of how these genotypes may relate to health or performance outcomes over time. Consequently, these data are best interpreted as a descriptive survey of allele frequencies in a specific Northern Chinese urban pediatric convenience sample and should not be generalized to guide population-level health or education policy. These limitations underscore that the data presented are descriptive in nature and are intended to serve as a baseline for future hypothesis-driven studies with richer phenotypic characterization.

### 4.8. Future Directions

Future research should include: longitudinal cohort studies tracking genotype–phenotype associations in the context of the new physical education policy, combining genotyping with objectively measured fitness outcomes and biochemical markers; expanded genotyping panels (e.g., ACE I/D, PPARGC1A, PPARD); studies examining whether genetic information, when integrated with comprehensive phenotypic characterization, can contribute to understanding individual differences in response to physical activity and nutritional interventions; and replication in geographically diverse Chinese populations to assess broader generalizability [36,43].

## 5. Conclusions

This cross-sectional study of 564 schoolchildren from two schools in two districts of Beijing provides genotype frequency data for four exercise- and folate metabolism-related genetic polymorphisms in this age group. The cumulative variant allele distribution across all four loci—with 87.23% of participants carrying at least two variant alleles and 29.26% carrying four or more—documents the frequency of genotypes previously associated with reduced folate metabolism enzyme activity in the literature. The diverse ACTN3 R577X genotype distribution documents the range of genetic variation at this locus in Beijing schoolchildren. These descriptive frequency data establish a baseline for future genotype–phenotype association studies; however, studies incorporating fitness, biochemical, and dietary data are required before such genetic profiles can inform educational or clinical recommendations. Given the purely descriptive, cross-sectional nature of the study and the absence of phenotypic data, these genotype frequency data do not, on their own, provide a basis for individualized physical education programming or nutritional recommendations.

## Figures and Tables

**Table 1:** PCR primer sequences.

Locus	Forward (5′–3′)	Reverse (5′–3′)
MTHFR C677T	CTCACCTGGATGGGAAAGAT	TCATCCCTATTGGCAGGTTAC
MTHFR A1298C	CACTCCAGCATCACTCACT	GGAGCTGAAGGACTACTACC
MTRR A66G	GTGATGAGGAGGTTTCTGTTACT	AACCTTATCGGATTCACTAATACAG
ACTN3 R577X	CACTGCTGCCCTTTCTGTTG	AGGGTGGTCACAGTATGCAG

**Table 2:** Genotype and allele frequencies for four polymorphisms in 564 Beijing schoolchildren.

Polymorphism	Genotype	*n* (%)	Allele	Freq. (95% CI)	HWE *p*
ACTN3 R577X	RR	178 (31.56)	R	0.561 (0.532–0.590)	0.932
(rs1815739)	RX	277 (49.11)	X	0.439 (0.410–0.468)	
	XX	109 (19.33)			
MTHFR C677T	CC	105 (18.62)	C	0.421 (0.392–0.451)	0.389
(rs1801133)	CT	265 (46.99)	T	0.579 (0.549–0.608)	
	TT	194 (34.40)			
MTHFR A1298C	AA	405 (71.81)	A	0.850 (0.828–0.870)	0.507
(rs1801131)	AC	149 (26.42)	C	0.150 (0.130–0.172)	
	CC	10 (1.77)			
MTRR A66G	AA	317 (56.21)	A	0.741 (0.715–0.766)	0.124
(rs1801394)	AG	202 (35.82)	G	0.259 (0.234–0.286)	
	GG	45 (7.98)			

HWE: Hardy-Weinberg equilibrium (exact test, Wigginton et al. [25]); CI: confidence interval.

**Table 3:** Sex-stratified genotype frequencies.

Polymorphism	Genotype	Male (n=265)	Female (n=299)	*p*
ACTN3 R577X	RR	86 (32.45)	92 (30.77)	0.700
	RX	125 (47.17)	152 (50.84)	
	XX	54 (20.38)	55 (18.39)	
MTHFR C677T	CC	50 (18.87)	55 (18.39)	0.971
	CT	125 (47.17)	140 (46.82)	
	TT	90 (33.96)	104 (34.78)	
MTHFR A1298C	AA	186 (70.19)	219 (73.24)	0.674
	AC	74 (27.92)	75 (25.08)	
	CC	5 (1.89)	5 (1.67)	
MTRR A66G	AA	154 (58.11)	163 (54.52)	0.636
	AG	91 (34.34)	111 (37.12)	
	GG	20 (7.55)	25 (8.36)	

Values are *n* (%) unless otherwise stated. *p* values from $\chi^{2}$ test.

**Table 4:** Allele frequency comparison with East Asian reference populations.

Polymorphism	Allele	Present Study	1000G CHB	*p*	gnomAD EAS	*p*
ACTN3 R577X	X	0.439	0.418	0.560	0.459	0.322
MTHFR C677T	T	0.579	0.563	0.682	0.531	0.013 ^a^
MTHFR A1298C	C	0.150	0.117	0.238	0.169	0.233
MTRR A66G	G	0.259	0.243	0.643	0.252	0.114

^a^ Borderline significant; did not survive Bonferroni correction (adjusted α=0.0125). gnomAD *p*-values are approximate (based on published frequencies). Abbreviations: CHB, Han Chinese in Beijing; EAS, East Asian.

**Table 5:** Multi-locus cumulative variant allele distribution across all four loci.

Allele Count	*n* (%)	Category
0	31 (5.50)	0–1 alleles
0	11 (1.95)	0–2 alleles
1	61 (10.82)	
2	145 (25.71)	
3	182 (32.27)	3–5 alleles
4	118 (20.92)	
5	42 (7.45)	
6	5 (0.89)	6–8 alleles
7	0 (0.00)	
8	0 (0.00)	

Variant alleles: ACTN3 X, MTHFR 677T, MTHFR 1298C, MTRR 66G. Allele count groups: 0–2, 3–5, 6–8. Allele counts of 7 and 8 were not observed.

**Table 6:** MTHFR C677T × MTHFR A1298C compound genotype distribution.

MTHFR C677T	MTHFR A1298C			Total
	AA	AC	CC	
CC	45 (7.98)	50 (8.87)	10 (1.77)	105 (18.62)
CT	168 (29.79)	97 (17.20)	0 (0.00)	265 (46.99)
TT	192 (34.04)	2 (0.35)	0 (0.00)	194 (34.40)
Total	405 (71.81)	149 (26.42)	10 (1.77)	564 (100)

Values are *n* (%). The 677TT + 1298CC combination was not observed, consistent with negative linkage disequilibrium.

**Table 7:** Pairwise linkage disequilibrium and haplotype estimates for MTHFR C677T and A1298C.

Measure	Value	95% CI
$|D^{\prime }|$	0.97	0.93–1.00
$r^{2}$	0.23	0.19–0.27

**Table 8:** Estimated MTHFR C677T–A1298C haplotype frequencies.

Haplotype	Frequency	SE
C-A	0.274	0.015
T-A	0.576	0.016
C-C	0.147	0.011
T-C	0.002	—

SE: standard error of the EM estimate. Haplotype notation: 677 allele-1298 allele.

**Table 9:** Multi-locus cumulative variant allele group distribution by sex.

Allele Count Group	Overall (n=564)	Male (n=265)	Female (n=299)	*p*
0–2 alleles	217 (38.48)	106 (40.00)	111 (37.12)	
3–5 alleles	342 (60.64)	156 (58.87)	186 (62.21)	0.637
6–8 alleles	5 (0.89)	3 (1.13)	2 (0.67)	

Variant alleles: ACTN3 X, MTHFR 677T, MTHFR 1298C, MTRR 66G. *p* value from $\chi^{2}$ test.

## Data Availability

The datasets generated and analyzed during the current study are available from the corresponding authors upon reasonable request.

## Supplementary Materials

The following supporting information can be downloaded at: https://www.mdpi.com/article/10.3390/genes17080886/s1, Table S1: Allele frequency comparison between Beijing schoolchildren and 1000 Genomes Phase 3 East Asian populations. Table S2: Four-locus compound genotype distribution. Table S3: Multi-locus linkage disequilibrium matrix for the four polymorphisms. Table S4: Sex-stratified multi-locus allele group distributions (extended). Table S5: Source data for the China MTHFR C677T geographical distribution map. Figure S1: Geographic distribution map of MTHFR C677T T allele frequency across China.

## Abbreviations

The following abbreviations are used in this manuscript: CHB, Han Chinese in Beijing; CI, confidence interval; EAS, East Asian; EM, expectation-maximization; gnomAD, Genome Aggregation Database; HWE, Hardy-Weinberg equilibrium; LD, linkage disequilibrium; MAF, minor allele frequency; MTHFR, methylenetetrahydrofolate reductase; MTRR, methionine synthase reductase; MVPA, moderate-to-vigorous physical activity; PCR, polymerase chain reaction.
