# Supplementary material for: Distribution of ACTN3 R577X, MTHFR C677T, MTHFR A1298C, and MTRR A66G Polymorphisms in 564 School-Aged Children in Beijing: A Cross-Sectional Study
Source: Genes (Basel). 2026 Jul 29;17(8):886. doi: 10.3390/genes17080886 (PMC13512591; doi:10.3390/genes17080886)
Supplement: Supplementary file 1 [file genes-17-00886-s001.zip › Figure_S1.pdf]

China MTHFR C677T T Allele Frequency Distribution

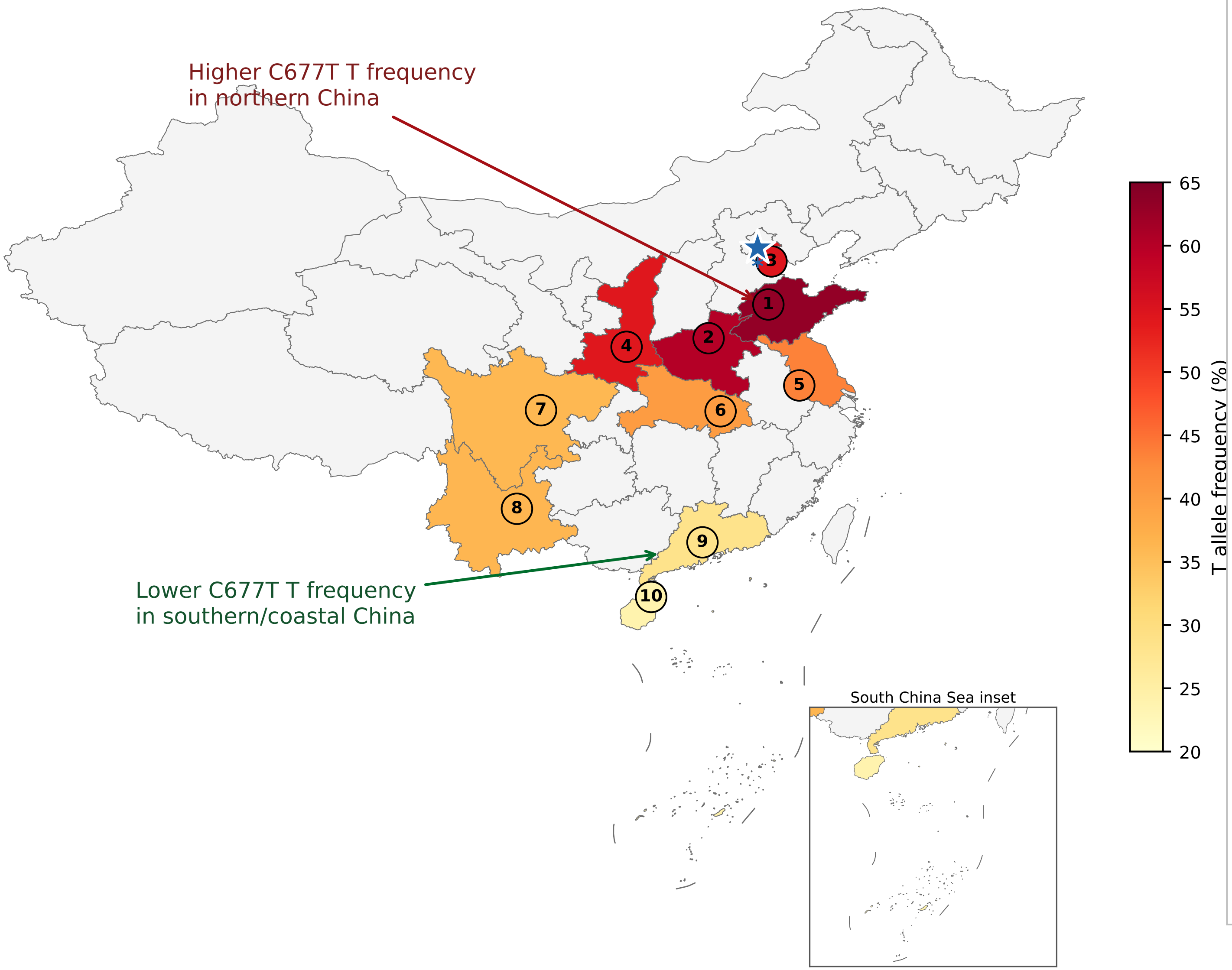

Mapped data points

No in-map labels are used to avoid overlap.

ID	Region	N	T%
1	Shandong	1052	63.1
2	Henan	2661	60.2
3	Tianjin	932	54.5
4	Shaanxi	3090	54.3
5	Jiangsu	477	43.5
6	Hubei	475	40.3
7	Sichuan	2108	36.0
8	Yunnan	124	36.3
9	Guangdong	470	28.5
10	Hainan	3016	24.0
★	**Beijing schoolchildren**	564	57.9

Latitude-frequency r = 0.914
(public 10-region data)

Primary source: Yang et al. PLoS One 2013, Table 1; PMID 23472119. Blue star: this study.

Boundary note: research visualization based on public province-level boundary data. Formal publication should follow MNR standard-map review requirements.
