# Supplementary material for: Distribution of ACTN3 R577X, MTHFR C677T, MTHFR A1298C, and MTRR A66G Polymorphisms in 564 School-Aged Children in Beijing: A Cross-Sectional Study
Source: Genes (Basel). 2026 Jul 29;17(8):886. doi: 10.3390/genes17080886 (PMC13512591; doi:10.3390/genes17080886)
Supplement: Supplementary file 1 [file genes-17-00886-s001.zip › Supplementary_Materials.pdf]

Supplementary Materials

Distribution of ACTN3 R577X, MTHFR C677T, MTHFR A1298C, and MTRR A66G Polymorphisms in 564 School-Aged Children in Beijing: A Cross-Sectional Study

This document contains supplementary figures and tables referenced in the main manuscript. Individual data files (.xlsx for tables, .png/.pdf for figures) are also available in the supplementary ZIP archive.

Contents:

1. Table S1. Allele frequency comparison between Beijing schoolchildren and 1000 Genomes Phase 3 East Asian populations
2. Table S2. Four-locus compound genotype distribution
3. Table S3. Multi-locus linkage disequilibrium matrix for the four polymorphisms
4. Table S4. Sex-stratified multi-locus allele group distributions (extended)
5. Table S5. Source data for the China MTHFR C677T geographical distribution map
6. Figure S1. Geographic distribution map of MTHFR C677T T allele frequency across China

Table S1. Allele frequency comparison between Beijing schoolchildren and 1000 Genomes Phase 3 East Asian populations

*Populations: CHB = Han Chinese in Beijing; CHS = Southern Han Chinese; CDX = Chinese Dai in Xishuangbanna; JPT = Japanese in Tokyo; KHV = Kinh in Ho Chi Minh City; EAS = East Asian combined; ALL = global. Significance: * p < 0.05, ** p < 0.01, *** p < 0.001.*

**Supplementary Table S1. Allele Frequency Comparison between Beijing Schoolchildren (n=564) and 1000 Genomes Phase 3 Populations**											
Frequencies on coding strand. Significance: * p<0.05, ** p<0.01, *** p<0.001											
ACTN3 R577X (rs1815739) | chr11:66560624 | Ref=R, Alt=X | Strand:+1 | Forward: C>T											
Population	N (indiv)	Alleles (n)	Ref count	Alt count	Alt freq	95% CI (low)	95% CI (high)	Chi-sq (df=1)	p-value	Signif	
Beijing schoolchildren (this study)	564	1128	633	495	0.4388	0.4096	0.4684	--	--	--	
Han Chinese in Beijing (CHB)	103	206	119	87	0.4223300970873786	0.3569061625692675	0.4905978595121499	0.1928159255450775	0.6605832085083871	ns	
Southern Han Chinese	105	210	126	84	0.4	0.3361096745159969	0.4674832653048799	1.087412171786648	0.2970452999808367	ns	

(CHS)											
Chinese Dai in Xishuangbanna (CDX)	93	186	92	94	0.5053763440860215	0.4341452509585357	0.5763898477764097	2.859007451957782	0.09086354943074777	ns	
Japanese in Tokyo (JPT)	104	208	107	101	0.4855769230769231	0.4185341513939457	0.5531427996864843	1.553141154533415	0.2126723198587733	ns	
Kinh in Ho Chi Minh City (KHV)	99	198	117	81	0.4090909090909091	0.342969224706225	0.4786730928338658	0.6062939434235977	0.4361866280063879	ns	
East Asian combined (EAS)	504	1008	561	447	0.4434523809523809	0.4130555341981196	0.474278609802285	0.04614101035835674	0.8299196769000504	ns	
Global (ALL)	2504	5008	3001	2007	0.400758785942492	0.3872671435327267	0.4144025660606492	5.525545263113266	0.01874076123766652	*	
MTHFR C677T (rs1801133) | chr1:11796321 | Ref=C, Alt=T | Strand:-1 | Forward: G>A (=coding C>T)											
Population	N (indiv)	Alleles (n)	Ref count	Alt count	Alt freq	95% CI (low)	95% CI (high)	Chi-sq (df=1)	p-value	Signif	
Beijing schoolchildren (this study)	564	1128	475	653	0.5789	0.5495	0.6079	--	--	--	
Han Chinese in Beijing (CHB)	103	206	110	96	0.4660194174757282	0.399143107521422	0.534139902139198	9.014432829610623	0.002678559939637198	**	
Southern Han Chinese (CHS)	105	210	150	60	0.2857142857142857	0.2288919234865963	0.3502358047009968	61.13679559377091	5.324367498661724e-15	***	
Chinese Dai in Xishuangbanna (CDX)	93	186	161	25	0.1344086021505376	0.09272482029439132	0.1908884657293253	126.3160994839773	2.622077903773235e-29	***	
Japanese in Tokyo (JPT)	104	208	129	79	0.3798076923076923	0.3165934502385702	0.4473811420983458	28.10236876202696	1.150649388958577e-07	***	
Kinh in Ho Chi Minh City (KHV)	99	198	160	38	0.1919191919191919	0.1431376082676703	0.2524280233959709	101.0756471079988	8.853720128816142e-24	***	

East Asian combined (EAS)	504	1008	710	298	0.2956449206349206	0.2682826261704738	0.3245390170590593	172.9259874973843	1.698762718303113e-39	***	
Global (ALL)	2504	5008	3779	1229	0.2454073482428115	0.2336869000398128	0.2575180893596926	481.5219082002184	9.967377968006817e-107	***	
MTHFR A1298C (rs1801131) | chr1:11794419 | Ref=A, Alt=C | Strand:-1 | Forward: T>G (=coding A>C)											
Population	N (indiv)	Alleles (n)	Ref count	Alt count	Alt freq	95% CI (low)	95% CI (high)	Chi-sq (df=1)	p-value	Signif	
Beijing schoolchildren (this study)	564	1128	959	169	0.1498	0.1295	0.172	--	--	--	
Han Chinese in Beijing (CHB)	103	206	160	46	0.2233009708737864	0.1717908586332507	0.2849422200317995	6.956451480512916	0.008352218235842486	**	
Southern Han Chinese (CHS)	105	210	177	33	0.1571428571428571	0.1141356964592218	0.2124686686409271	0.07401098846078366	0.7855838766543324	ns	
Chinese Dai in Xishuangbanna (CDX)	93	186	132	54	0.2903225806451613	0.2298562447876442	0.3592749045494875	22.36864807123759	2.25017463896672e-06	***	
Japanese in Tokyo (JPT)	104	208	171	37	0.1778846153846154	0.1318982360910953	0.2355536713718393	1.060395812618439	0.3031249266460386	ns	
Kinh in Ho Chi Minh City (KHV)	99	198	147	51	0.2575757575757576	0.2016841341829498	0.3226953792572925	14.1318575509768	0.0001704328100357139	***	
East Asian combined (EAS)	504	1008	787	221	0.2192460317460317	0.1947965719573445	0.2458273359744898	17.18988616040601	3.382322936356723e-05	***	
Global (ALL)	2504	5008	3759	1249	0.2494009584664537	0.2376127823869875	0.2615733052180158	51.37522062020965	7.62955056654793e-13	***	
MTRR A66G (rs1801394) | chr5:7870860 | Ref=A, Alt=G |											

Strand:+1 | Forward: A>G											
Population	N (indiv)	Alleles (n)	Ref count	Alt count	Alt freq	95% CI (low)	95% CI (high)	Chi-sq (df=1)	p-value	Signif	
Beijing schoolchildren (this study)	564	1128	836	292	0.2589	0.2335	0.2855	--	--	--	
Han Chinese in Beijing (CHB)	103	206	154	52	0.2524271844660194	0.198008361242079	0.3159107091424395	0.03772672092258186	0.845992915262649	ns	
Southern Han Chinese (CHS)	105	210	157	53	0.2523809523809524	0.1984388234492946	0.3152198846785909	0.03889930185276052	0.8436482911441994	ns	
Chinese Dai in Xishuangbanna (CDX)	93	186	139	47	0.2526881720430108	0.1956740718158342	0.3197113863766801	0.03182577664316721	0.8584105734203116	ns	
Japanese in Tokyo (JPT)	104	208	145	63	0.3028846153846154	0.2444772766113502	0.3684410548211919	1.744090922082471	0.1866213135749185	ns	
Kinh in Ho Chi Minh City (KHV)	99	198	148	50	0.2525252525252525	0.1971127094777315	0.3173580438258491	0.03537321521370903	0.8508157545107556	ns	
East Asian combined (EAS)	504	1008	743	265	0.2628968253968254	0.2366579074797483	0.2909361349079139	0.04488316862929014	0.8322190028521708	ns	
Global (ALL)	2504	5008	3184	1824	0.3642172523961661	0.3509982307029682	0.3776444303101014	45.22750788678823	1.754237042449017e-11	***	

Table S2. Four-locus compound genotype distribution

Beijing Schoolchildren (N = 564). Loci: ACTN3 R577X (RR/RX/XX), MTHFR C677T (CC/CT/TT), MTHFR A1298C (AA/AC/CC), MTRR A66G (AA/AG/GG). 50 out of 81 possible combinations observed. Sorted by descending frequency.

**Supplementary Table S2. Four-Locus Compound Genotype Distribution in Beijing Schoolchildren (N=564)**								
Loci: ACTN3 R577X (RR/RX/XX), MTHFR C677T (CC/CT/TT), MTHFR A1298C (AA/AC/CC), MTRR A66G (AA/AG/GG)								
50 out of 81 possible (3x3x3x3) combinations observed. Sorted by descending frequency.								
Rank	ACTN3	MTHFR C677T	MTHFR A1298C	MTRR	N	%	Cumul %	Variant Alleles
1	RX	TT	AA	AA	52	9.24	9.24	3
2	RX	TT	AA	AG	40	7.1	16.34	4
3	RX	CT	AA	AA	40	7.1	23.45	2
4	RR	TT	AA	AA	36	6.39	29.84	2
5	RR	CT	AA	AA	33	5.86	35.7	1
6	RX	CT	AA	AG	31	5.51	41.21	3
7	XX	CT	AA	AA	25	4.44	45.65	3
8	RX	CT	AC	AA	19	3.37	49.02	3
9	RX	CT	AC	AG	18	3.2	52.22	4
10	RR	CT	AA	AG	18	3.2	55.42	2
11	RR	CT	AC	AA	17	3.02	58.44	2
12	XX	TT	AA	AA	16	2.84	61.28	4
13	RR	TT	AA	AG	16	2.84	64.12	3
14	RX	CC	AA	AA	15	2.66	66.79	1
15	XX	TT	AA	AG	15	2.66	69.45	5
16	XX	CT	AC	AA	14	2.49	71.94	4
17	RR	CT	AC	AG	13	2.31	74.25	3
18	RX	CC	AC	AA	12	2.13	76.38	2
19	RR	CC	AA	AA	11	1.95	78.33	0
20	RX	CC	AC	AG	10	1.78	80.11	3
21	RX	TT	AA	GG	10	1.78	81.88	5
22	RR	CC	AC	AA	9	1.6	83.48	1
23	RX	CT	AA	GG	9	1.6	85.08	4
24	XX	CT	AA	AG	9	1.6	86.68	4
25	RR	CC	AC	AG	8	1.42	88.1	2
26	XX	CT	AC	AG	7	1.24	89.34	5
27	XX	CC	AC	AA	6	1.07	90.41	3
28	RX	CC	AA	AG	6	1.07	91.47	2

29	RR	TT	AA	GG	5	0.89	92.36	4
30	RX	CT	AC	GG	5	0.89	93.25	5
31	XX	CC	AA	AA	5	0.89	94.14	2
32	RR	CC	AA	AG	4	0.71	94.85	1
33	RX	CC	CC	AA	4	0.71	95.56	3
34	XX	CT	AC	GG	3	0.53	96.09	6
35	RX	CC	CC	AG	2	0.36	96.45	4
36	XX	TT	AA	GG	2	0.36	96.8	6
37	RR	CC	CC	AA	2	0.36	97.16	2
38	RR	CT	AA	GG	2	0.36	97.51	3
39	RX	CC	AC	GG	2	0.36	97.87	4
40	XX	CC	AA	AG	2	0.36	98.22	3
41	XX	CC	AC	GG	1	0.18	98.4	5
42	XX	CT	AA	GG	1	0.18	98.58	5
43	RR	CC	AA	GG	1	0.18	98.76	2
44	RR	CC	AC	GG	1	0.18	98.93	3
45	XX	CC	CC	AG	1	0.18	99.11	5
46	XX	CC	AC	AG	1	0.18	99.29	4

Note: All 564 individuals had complete genotype data for all four loci. No individuals were excluded due to missing data. Variant allele count range: 0–8.

Table S3. Multi-locus linkage disequilibrium matrix for the four polymorphisms

All pairwise LD statistics among 4 polymorphisms in Beijing schoolchildren (N = 564). Method: EM algorithm from unphased genotype data. MTHFR677×MTHFR1298 (chromosome 1, ~2 kb apart) show strong LD. Cross-chromosome pairs have $|D'| < 0.12$ *and* $r^2 < 0.004$.

**Supplementary Table S3. Multi-Locus Linkage Disequilibrium Matrix (all pairwise comparisons)**										
LD statistics estimated via EM algorithm from unphased genotype data (N=564). D' and r2 matrices for 4 loci.										
Table 1. |D'matrix (standardized linkage disequilibrium coefficient)										
	ACTN3	MTHFR677	MTHFR1298	MTRR						

ACTN3	1	0.02726822171869664	0.007792742778008492	0.02567613117776351						
MTHFR677	0.02726822171869664	1	0.971387978457371	0.1096223572605695						
MTHFR1298	0.007792742778008492	0.971387978457371	1	0.03837289281682253						
MTRR	0.02567613117776351	0.1096223572605695	0.03837289281682253	1						
Table 2. r2 matrix (correlation coefficient between alleles)										
	ACTN3	MTHFR677	MTHFR1298	MTRR						
ACTN3	1	0.0004194737629126164	1.357798540079223e-05	0.000295000312370817						
MTHFR677	0.0004194737629126164	1	0.226848208330587	0.003033561097680473						
MTHFR1298	1.357798540079223e-05	0.226848208330587	1	0.0007357672140121302						
MTRR	0.000295000312370817	0.003033561097680473	0.0007357672140121302	1						
Table 3. Detailed pairwise LD statistics										
Locus 1	Locus 2	Chr 1	Chr 2	Ref allele 1 freq	Ref allele 2 freq	D	|D'|	r2	Chi-sq (df=1)	p-value
ACTN3 R577X	MTHFR C677T	11	1	0.5621669626998224	0.4200710479573712	0.005015198137957616	0.02726822171869664	0.0004194737629126164	0.9446549140792122	0.5621669626998224
ACTN3 R577X	MTHFR A1298C	11	1	0.5621669626998224	0.8516873889875666	0.0006497312290839607	0.007792742778008492	1.357798540079223e-05	0.03057762312258411	0.5621669626998224

ACTN3 R577X	MTRR A66G	11	5	0.5621669626998224	0.7415640550621669	0.0037303493333324057	0.02567613117776351	0.000295000312370817	0.6643407034590799	0.5621669626998224
MTHFR C677T	MTHFR A1298C	1	1	0.4200710479573712	0.8516873889875666	-0.08354983487245976	0.971387978457371	0.226848208330587	510.8621651604819	0.4200710479573712
MTHFR C677T	MTRR A66G	1	5	0.4200710479573712	0.7415640550621669	0.01190080900688756	0.1096223572605695	0.003033561097680473	6.831579591976425	0.4200710479573712
MTHFR A1298C	MTRR A66G	1	5	0.8516873889875666	0.7415640550621669	0.004220371738909656	0.03837289281682253	0.0007357672140121302	1.656947765955317	0.8516873889875666
Method: Haplotype frequencies estimated by EM algorithm from unphased two-locus genotype counts. |D'| = D/Dmax, where D = h_ij - p_i*q_j; r2 = D2/(pi(1-pi)qj(1-qj)). Chi-square test with df=1: chi2 = 2N*r2.										
Interpretation: MTHFR										

C677T and A1298C (both on chr1) show strong LD (|D'|=0.9714, r2=0.2268), consistent with close physical proximity (~2 kb apart). Cross-chromosome pairs show |D'| < 0.12 and r2 < 0.004, consistent with linkage equilibrium except for a weak MTHFR C677T-MTRR A66G signal (p=0.009).										

Table S4. Sex-stratified multi-locus allele group distributions (extended)

N = 564 (Male = 265, Female = 299). Variant alleles: ACTN3 X + MTHFR 677T + MTHFR 1298C + MTRR 66G (range 0–8). Group 0–2, Group 3–5, Group 6–8. No significant sex differences (all p > 0.05).

Supplementary Table S4. Sex-Stratified Multi-Locus Cumulative Variant Allele Count and Category Distribution							
Variant alleles summed across ACTN3 X, MTHFR 677T, MTHFR 1298C, MTRR 66G. Range: 0-8. N=564 (M=265, F=299)							
Table 1a. Individual Cumulative Variant Allele Count Distribution by Sex							
Variant Allele Count	Male N	Male %	Female N	Female %	Total N	Total %	Chi-sq P
0	6	2.26	5	1.67	11	1.95	0.6119
1	31	11.70	30	10.03	61	10.82	0.5252
2	69	26.04	76	25.42	145	25.71	0.8665
3	81	30.57	101	33.78	182	32.27	0.4153
4	52	19.62	66	22.07	118	20.92	0.4751
5	23	8.68	19	6.35	42	7.45	0.2939
6	3	1.13	2	0.67	5	0.89	0.5581
7	0	0	0	0	0	0	—
8	0	0	0	0	0	0	—
Table 1b. Variant Allele							

Group Distribution by Sex							
Category	Male N	Male %	Female N	Female %	Total N	Chi-sq (df=1)	p-value
0-2 alleles	106	40.00	111	37.12	217	—	0.4835
3-5 alleles	156	58.87	186	62.21	342	—	0.4178
6-8 alleles	3	1.13	2	0.67	5	—	0.5581

Table S5. Source data for the China MTHFR C677T geographical distribution map

Data from Yang et al. 2013 PLoS One Table 1 (PMID: 23472119) plus the present Beijing schoolchildren cohort. Latitude-frequency Pearson r = 0.914. Map ID (*) = this study.

**Supplementary Table S5. Data Used for China MTHFR C677T Geographical Distribution Map**															
T allele frequency is reported as percent. Current figure uses numbered non-overlapping points and a right-side legend table.															
Map ID	Region	Province/municipality	Macro-region	Latitude	Longitude	N	CC	CT	TT	C allele count	T allele count	T frequency (%) source	T frequency (%) recalculated	TT genotype (%)	95% CI/source note
1	Shandong	山东省	Northern	36.67	117.02	1052	154	469	429	777	1327	63.1	63.07	40.78	60.9-65.1
2	Henan	河南省	Northern	34.75	113.62	2661	441	1236	984	2118	3204	60.2	60.2	36.98	58.9-61.5
3	Tianjin	天津市	Northern	39.12	117.2	932	199	450	283	848	1016	54.5	54.51	30.36	53.1-55.6
4	Shaanxi	陕西省	Northern	34.26	108.95	3090	670	1482	938	2822	3358	54.3	54.34	30.36	52.2-56.8
5	Jiangsu	江苏省	Southern	32.06	118.78	477	156	227	94	539	415	43.5	43.5	19.71	40.3-46.7
6	Hubei	湖北省	Southern	30.59	114.3	475	172	223	80	567	383	40.3	40.32	16.84	37.2-43.5
7	Sichuan	四川省	Southern	30.65	104.08	2108	882	936	290	2700	1516	36	35.96	13.76	34.5-37.4
8	Yunnan	云南省	Sout	25.0	102.7	12	53	52	1	15	90	36.3	36.29	15.32	30.3-

			hern	4	1	4			9	8					42.6
9	Guangdong	广东省	Southern	23.13	113.27	470	241	190	39	672	268	28.5	28.51	8.3	25.6-31.5
10	Hainan	海南省	Southern	20.02	110.35	3016	1763	1061	192	4587	1445	24	23.96	6.37	24.6-26.8 (source CI appears inconsistent; transcribed verbatim)
*	Beijing schoolchildren	北京市	This study	39.9	116.41	564	105	265	194	475	653	57.89	57.89	34.4	54.95-60.79

Figure S1. Geographic distribution map of MTHFR C677T T allele frequency across China

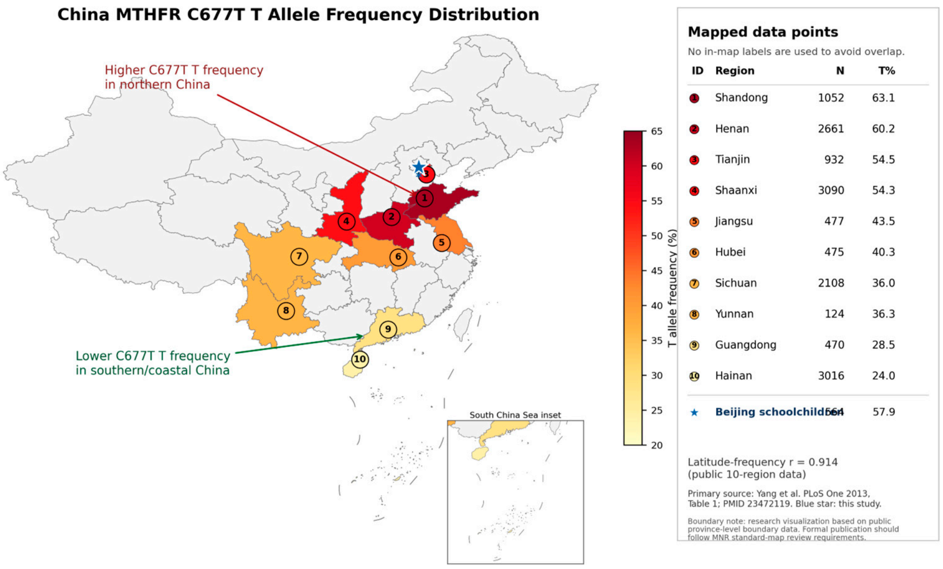

Numbered points correspond to Map IDs in Table S5. Latitude–frequency Pearson r across 10 literature regions: 0.914. For formal publication in China, verify or replace the base map using the Ministry of Natural Resources standard map service per journal requirements.
